# Editorial

**Published:** 1844-09-21

**Authors:** 


### THE MEDICAL EXAMINER.

### PHILADELPHIA, SEPT. 21, 1844.

### NEW YORK JOURNAL OF MEDICINE.

What may be the particular connexion existing between the editor of that journal and Chailly's Midwifery, we know not, but it seems always present to his eyes, like Hamlet's ghost, beckoning him on to some terrible revenge. In his last number (September, 1844) he continues to assert that Chailly's Midwifery has "encountered opposition," as he is pleased to say of our strictures, *only* in the Examiner—with how much truth, the following extracts will show:

"While, however, we accord what we believe to be its due meed of praise to the treatise of Mr. Chailly, we cannot subscribe to the extravagant eulogium bestowed upon it in the preface of the American editor. It certainly cannot, with any degree of propriety, be said to "combine *all that is new and valuable in obstetric science.*" * * * * * "It is neither a complete treatise of Midwifery, nor does it embrace a full and faithful exposition of the views of every authoritative writer on all points connected with the principles and practice of the obstetric art; and moreover, were we to enter into a more particular examination of each portion of the work, we should feel it our duty to call in question the accuracy of some of the views and practical directions of the author.

Upon the whole, even for the use of the young student, we should prefer the work of Rigby, and for those more advanced, we have many, in all respects, superior; while, as a work of reference for the practitioner, the treatise of Churchill will be found much more full and satisfactory.

The two hundred and sixteen wood cuts introduced as illustrations of the text, and which, the author assures us, he designed himself, are among the most clumsy with which we are acquainted. They certainly do very little credit to the author's skill as a draftsman; and, while they are far from being ornamental, they are, in one or two instances, of very questionable accuracy."—*Amer. Jour. of the Medical Sciences, July*, 1844, *page* 189.

"Much has been done of late years to elevate the art of Midwifery to the station it deserves. In Germany, Naegele, in Great Britain, Rigby, Ramsbotham, Churchill and others, and in our own country Dewees, Meigs, Hodge, &c., have contributed valuable quota. The student cannot complain of a dearth of matter on this subject. Valuable books on Obstetrics are abundant, and now another work appears. But we cannot say that its addition to the stock brings any improvement. The details are meagre. No reference is made to the English or American authorities. The German writers are not quoted, save an occasional reference to Professor Naegele. The French works are alone appealed to, and every one knows that France has been, of late, singularly deficient in obstetrical improvement. The defect has not been supplied by Dr. Bedford in his notes, which are not very valuable. On the whole, the work is not by any means to be compared to the treatises of various authors already before the public."—*St. Louis Med. and Sur. Jour. July* 1, 1844.

Beside these notices of the work, some of its editorial matter has been severely animadverted on by a correspondent of the Boston Medical and Surgical Journal, in some of its late numbers.

All of these criticisms, be it known, had appeared in the Medical journals of the country weeks or months before the New York editor's last publication. Comment is unnecessary.

As to the unworthy suggestion of the editor of the N. Y. Journal, that we are *prompted* to "special opposition" to him, it is altogether idle. We have no such promptings nor any such feeling. Let him cease to abuse and misrepresent us, and whenever he shall find occasion to remark on anything we may write, do it with proper temper, and in language befitting a Medical Journal, and he will experience no "special opposition" on our part.

---

### ARTIFICIAL ABORTION.

We extract the following strange statement and remarks of the editor, from the Boston Medical and Surgical Journal of the 11th inst.

"*Artificial Abortions.*—A singular doctrine, we are informed, has been taught here within a few weeks, which strangely conflicts with the public sentiment, and the supposed experience of medical men. A worthless, itinerating quack having been accused of producing an abortion, as already mentioned in the Journal, being apprehensive of an arrrest, fled from the city. Preparations for having the offender indicted were making, when, in order to render doubly sure what was already thought a sure case, the opinion of some magnates in the profession was sought, by a legal gentleman, as to the means resorted to, usually, to bring about premature expulsion of the fœtus. To the surprise of the inquirer, it is asserted that they unhesitatingly decided that abortion could not be affected by medicine!

Here the thing will rest for the present. But notwithstanding this learned opinion, so very contrary to the general belief of physicians, many fully believe that there are half a dozen unprincipled scoundrels, at least, in Boston, whose fees for accomplishing just what is declared to be impossible, are surprisingly large. These fellows are laughing in their sleeves at such a decision, which their daily iniquity contradicts, and which will hereafter be more openly conducted, since some doctors say the thing is impossible!

Abortions are probably quite as frequently brought about by drugs as instruments; but the perpetrators of these fœtal murders have always managed adroitly to escape punishment. Hereafter, they will be under no restraining fear, since by high and unquestioned authority it is established *that they cannot do what they are constantly doing* in violation of the laws of the land and the higher laws of God.

The Journal is open for the record of facts and for the discussion of this extraordinary doctrine, which is likely to have a pretty close investigation."

Every one must regret that the names of the "*magnates*" who gave such an opinion are not mentioned, as it is difficult to understand how any intelligent and experienced practitioner could seriously assert such a doctrine. That all attempts by evil disposed persons to effect abortion by the administration of drugs, frequently fail, we know by experience, and that such a result can not be accomplished without considerable risk to the life of the woman, is unquestionable; but that any one eminent in the profession should assert it to be *impossible*, seems to us incredible. Abortion, we conceive, is *never natural*"—it is the consequence of some morbid condition of the uterus, or some great disturbing agency; and do not various medicines, in large doses, cause a sufficient disturbance to interfere seriously with the various functions of the body? Certainly they do; and accordingly it is not a very unusual circumstance for abortion to follow the use of cathartics, ergot, &c., administered for a different purpose, as we presume most physicians of experience must have witnessed.

---

The editor of the *New York Journal of Medicine* speaks of the *Medical Examiner* as a "pop gun," and *his* journal as a "big gun—a Paixhan—a 'peace maker'"! Well, possibly there may be some analogy—let us see. The "big gun—the Paixhan—the peacemaker," (we like to be particular) made a great noise! but, like Uncle Toby's armies, that "swore terribly in Flanders," it killed no enemy! Let out cotemporary ponder on this. It is a dangerous thing to become too "big"—a *burst* is very apt to follow.

